# The therapeutic efficacy of Chinese patent medicine combined with routine western medicine in the treatment of coronavirus disease 2019 (COVID-19): A protocol for systematic review and meta-analysis

**DOI:** 10.1097/MD.0000000000022277

**Published:** 2020-09-18

**Authors:** Jingxia Zhang, Shasha Li, Chongbo Zhao, Weifeng Wang, Fan Li, Fang Li

**Affiliations:** a Pharmacy College, Shaanxi University of Chinese Medicine, Xianyang; b Shaanxi Academy of Traditional Chinese Medicine, Xi’an, Shaanxi, China.

**Keywords:** Chinese patent medicine, corona virus disease 2019, meta-analysis, protocol, routine western medicine, systematic review

## Abstract

**Background:**

Coronavirus disease 2019 (COVID-19) is a potentially fatal disease. clinical practice shows that Chinese Patent Medicine (CPM) has played an important role in the outbreak, Among them, Jinhua Qinggan granules, Lianhua Qingwen capsule, and Xuebiqing injection have an effect in treating COVID-19 patients, but it has not been systematically evaluated for efficacy and safety. We provide a protocol for systematic review and meta-analysis.

**Materials and methods:**

Retrieved the database, including the China National Knowledge Infrastructure, Chinese Biomedical Database, Wan Fang database, and PubMed. The quality of each study is assessed according to the criteria of the Cochrane Handbook for Systematic Reviews of Interventions. Using Manager 5.3 software and STATA 16.0 software were used to perform the meta-analysis.

**Results:**

The systematic review and meta-analysis aims to review and pool current clinical outcomes of CPM combined with routine western medicine (RWM) for the treatment of COVID-19.

**Conclusion:**

This study will provide evidence of CPM (including Jinhua Qinggan granule, Lianhua Qingwen capsule, and Xuebiqing injection) for the treatment on COVID-19 patients.

INPLASY Registration number: INPLASY202050050.

## 1 Introduction

Coronavirus disease 2019 (COVID-19) is a viral respiratory disease caused by the 2019 novel coronavirus (2019-nCoV), which has posed an enormous threat to public health. The COVID-19 known as the severe acute respiratory syndrome coronavirus 2 (SARS-CoV- 2), which is a newly emerging zoonotic agent and has caused the pneumonia epidemic in the world. As of August 7, 2020, the outbreak of COVID-19 has infected over 18,614,177 people globally, with nearly 702,642 deaths in the world reported by the World Health Organization (WHO), since its emergence in December 2019.^[1]^ Therefore, the effective prevention and treatment of COVID-19 are a very urgent task. The treatment of COVID-19 involves multiple disciplines, and the current recommendations are mainly based on Western Medicine including supportive care, anti-infection (mainly antiviral agents), and glucocorticoid therapy, but they cannot be used as special drugs for anti-COVID-19, prompting the need for novel treatment options.

The Traditional Chinese medicine (TCM) exhibited remarkable benefits against the prevention, treatment, and rehabilitation of COVID-19.^[2]^ CPM (including Jinhua Qinggan granule, Lianhua Qingwen capsule, and Xuebiqing injection) was recommended as the treatment agent in the 7th edition of the "Diagnosis and Treatment Scheme for New Coronavirus Infected Pneumonia”.^[3]^ There is now evidence that Traditional Chinese and Western Medicine have a significant effect on the treatment of COVID-19.^[4]^ First, Lianhua Qingwen is composed of Lianqiao, Jinyinhua, Mahuang, Kuxingren, Shigao, Banlangen, Mianmaguanzhong, Guanghuoxiang, Dahuang, Yuxingcao, Hongjingtian, Bohenao, Gancao. It combined with western medicine had considerable effective rate without obvious adverse reactions.^[2]^ In addition, clinical studies have shown that Lianhua Qingwen can treat A/H1N1 influenza and has a broad-spectrum antiviral effect.^[5]^ Second, Jinhua Qinggan is composed of Jinyinhua, Shigao, Mahuang, Kuxingren, Huangqin, Lianqiao, Zhebeimu, Zhimu, Niubangzi, Qinghao, Bohe, Gancao. It has a significant effect on the treatment of mild, common COVID-19, reflected in the reduction of fever time, improvement of symptoms and reduction of inflammation.^[6]^ Xuebijing Injection is composed of Honghua, Chishao, Chuanxiong, Danshen, Danggui, the prescription originated from therapeutic principles of TCM, which was approved as a second grade national new medicine for treating sepsis in China over 15 years.^[2]^ It has significant effect in clinical symptoms of patients and preventing the deterioration of the disease.^[7]^ However, because of comprehensive and systematic evidence, we will present a meta-analysis protocol of the therapeutic efficacy of CPM combined with RWM vs RWM on COVID-19.

## 2 Methods and program

The protocol has been registered on the International prospective register of systematic review (INPLASY), the registration number is INPLASY202050050. The protocol followed Preferred Reporting Items for Systematic review and Meta-Analysis Protocols (PRISMA-P) guidelines.^[8]^

### 2.1 Literature retrieval strategy

Keywords “Lianhua Qingwen, Jinhua Qinggan, Xuebijing injection or Xuebijing” [Title/Abstract] AND “COVID-19” [Title/Abstract] or “New coronavirus” [Title/Abstract] or “Novel coronavirus pneumonia” or “2019-nCoV” were used as search items in electronic databases including Pubmed, Wanfang, the China National Knowledge Infrastructure and Spring. Full-text review was performed while the title/abstract thought to be thematic. The job above was executed by 2 investigators independently. The conflicts were resolved by consensus and discussion.

### 2.2 Inclusion and exclusion criteria

We designed the inclusion criterion as follows:

1. The clinical trials involved were randomized controlled trials.
2. Meet the diagnostic criteria of COVID-19 mild patients issued by the National Health and Health Commission of the Peoples Republic of China on the diagnosis and treatment of new coronavirus infection pneumonia (trial version 7), for example, the specific IgM and IgG antibodies against SARS-COV-2 were positive in serum test.
3. Intervention group: the treatment group was given CPM on the basis of RWM treatment. Control group: only RWM treatment. RWM treatment includes symptomatic treatments such as antiviral and anti-infection, for example, oral lopinavir, ritonavir, etc.
4. Main outcomes: total efficacy, relief time of main symptoms (such as fever, cough, myalgia, or fatigue), relief time of other symptoms (such as headache, dizziness, diarrhea, nausea, and so on.). Additional outcomes: leukocyte, lymphocyte, C-reactive protein, adverse events.

An exclusion criterion was designed as follows:

1. Articles such as reviews, animal experiments, case report, and comments et al were thought to be unrelated with the topic.
2. Experiments without control or diagnostic criteria statement was ambiguous.
3. The intervention of COVID-19 patients was not based on Chinese Patent Medicine (including Lianhua Qingwen, Jinhua Qinggan, and Xuebijing injection) treatment.

**Figure 1:**
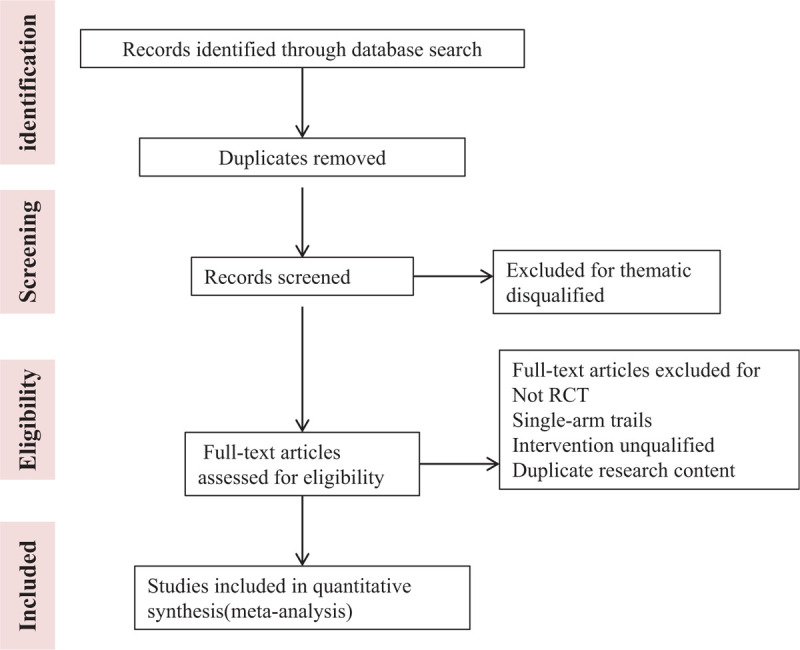
Process of study screening.

The full screening process is shown in PRISMA flow chart (Fig. 1).

### 2.3 Data extraction and quality assessment

The quality of included studies was assessed by 2 investigators independently according to the Cochrane Handbook for Systematic Reviews of interventions.^[4]^ Disagreement was resolved by the consensus. Quality assessment was evaluated as follows: random sequence generation (selection bias), allocation concealment (selection bias), blinding of participants and personnel (performance bias), blinding of outcome assessment (detection bias), incomplete outcome data (attrition bias), selective reporting (reporting bias) and other bias. Each term was judged with 3 levels. “Low risk” bias means the description of methods or procedures was adequate, “High risk” indicates the description of methods or procedures was not adequate or incorrect while “Unclear risk” bias means there was no description of methods and/or procedures.

### 2.4 Data analysis

In the process, 2, investigators will extract detailed information and available data from the qualified studies, such as sample size, intervention, duration of intervention, and outcome measures. If there are disagreements during the evaluation process, it will be resolved through discussion with a third investigator. Data analysis was performed using Review Manager 5.3 (Cochrane Collaboration) and STATA 16.0 software.

Outcome measures such as Total effective were regarded as dichotomous variables and presented as the odds ratio (OR) with 95% confidence intervals (95% CI), and the risk ratio (OR) with 95% confidence intervals (95% CI), Contents of inflammatory cytokines were continuous variables that presented as the mean difference (MD) with 95% CI, Q statistic and *I*^2^ tests were applied to assess the heterogeneity among studies. When *I*^2^ ≤ 25%, the data is considered homogeneous. A fixed-effects model was used to analyze data with low heterogeneity (25% ≤ *I*^2^ ≤ 50%) and data with high heterogeneity (*P* < .1 or *I*^2^ > 50%) was estimated using random-effects model. Potential publication bias was revealed by funnel plots.^[9,10]^

## 3 Discussion

The COVID-19 has rapidly spread around the world, The World Health Organization has declared the coronavirus disease (COVID-19) as a pandemic on 11 March 2020,^[11]^ and the COVID-19 infected pneumonia was deemed to the category of “Pestilence”. In the theory of traditional Chinese medicine, the characteristic of its pathogenesis were “dampness, toxin, stasis and closure”.^[2]^ Western medicines has some effects, but they cannot be used as special drugs for anti-COVID-19, So we need novel treatment options.

Chinese herbal formula is an alternative approach for prevention of COVID-19 in high-risk population, according to historical records and human evidence of SARS and H1N1 influenza prevention.^[12]^ CPMs (including Jinhua Qinggan granule, Lianhua Qingwen capsule, and Xuebiqing injection) can achieve considerable effects for both suspected cases under medical observation period and confirmed individuals with serious underlying diseases or critical conditions.^[2]^ Therefore, it is necessary to use this research protocol to explore the efficacy and safety of CPMs on COVID-19.

## Author contributions

**Methodology:** Jingxia Zhang.

**Project administration:** Jingxia Zhang.

**Resources:** Jingxia Zhang.

**Software:** Shasha Li, Fan Li.

**Supervision:** Fang Li.

**Validation:** Weifeng Wang.

**Visualization:** Weifeng Wang, Chongbo Zhao.

**Writing – original draft:** Jingxia Zhang, Shasha Li.

**Writing – review & editing:** Fang Li, Weifeng Wang.

## Abbreviations

Abbreviations: COVID-19 = corona virus disease 2019, CPM = Chinese Patent Medicine, RWM = routine western medicine.
